# Using Strategic Movement to Calibrate a Neural Compass: A Spiking Network for Tracking Head Direction in Rats and Robots

**DOI:** 10.1371/journal.pone.0025687

**Published:** 2011-10-04

**Authors:** Peter Stratton, Michael Milford, Gordon Wyeth, Janet Wiles

**Affiliations:** 1 Queensland Brain Institute, The University of Queensland, Brisbane, Queensland, Australia; 2 School of Information Technology and Electrical Engineering, The University of Queensland, Brisbane, Queensland, Australia; 3 School of Engineering Systems, Queensland University of Technology, Brisbane, Queensland, Australia; University of California Irvine, United States of America

## Abstract

The head direction (HD) system in mammals contains neurons that fire to represent the direction the animal is facing in its environment. The ability of these cells to reliably track head direction even after the removal of external sensory cues implies that the HD system is calibrated to function effectively using just internal (proprioceptive and vestibular) inputs. Rat pups and other infant mammals display stereotypical warm-up movements prior to locomotion in novel environments, and similar warm-up movements are seen in adult mammals with certain brain lesion-induced motor impairments. In this study we propose that synaptic learning mechanisms, in conjunction with appropriate movement strategies based on warm-up movements, can calibrate the HD system so that it functions effectively even in darkness. To examine the link between physical embodiment and neural control, and to determine that the system is robust to real-world phenomena, we implemented the synaptic mechanisms in a spiking neural network and tested it on a mobile robot platform. Results show that the combination of the synaptic learning mechanisms and warm-up movements are able to reliably calibrate the HD system so that it accurately tracks real-world head direction, and that calibration breaks down in systematic ways if certain movements are omitted. This work confirms that targeted, embodied behaviour can be used to calibrate neural systems, demonstrates that ‘grounding’ of modelled biological processes in the real world can reveal underlying functional principles (supporting the importance of robotics to biology), and proposes a functional role for stereotypical behaviours seen in infant mammals and those animals with certain motor deficits. We conjecture that these calibration principles may extend to the calibration of other neural systems involved in motion tracking and the representation of space, such as grid cells in entorhinal cortex.

## Introduction

### Overview

Calibration is a major issue for all real-world systems, animal and robotic. Of particular interest in this paper is how movement strategies of an animal or robot may combine with learning rules to calibrate a neural system. Strategic movements yield information that random movement does not, and embodiment on a physical system provides real world sensory input that is absent from disembodied neural systems. In robotics, practical methods for calibration of the fundamental components of navigation systems are essential. In this paper we are particularly interested in the head direction system, due to its foundational role in navigating systems, both mammalian and robotic.

Head direction (HD) neurons are so-named because they fire only when an animal is facing in specific directions relative to cues in the environment [1], [2]. Each HD neuron fires maximally for typically just one preferred head direction, with firing tapering off as the head turns away from this direction. In a population of HD neurons, all directions are represented approximately equally giving a unique activity pattern called the *bump* or *hill of activity* for any given direction the animal faces [3]–[8]. The peak of the bump represents the animal's current direction and those neurons which are firing to represent this direction will continue to fire at about the same rate for as long as the animal's direction remains the same [9]. When the animal moves, the bump translates in a systematic way through the HD neuron population such that the peak continues to represent the current head direction.

The HD system is thought to function as a *continuous attractor* neural network, allowing the system to represent any possible head direction [10]. Inherent in such attractor networks is a tendency to *drift* from any given state, since all adjoining attractor states (head directions) are equally stable and even minor perturbations or noise can cause a spontaneous shift to an adjoining state. Implicit in most existing models of HD networks is the assumption that the synaptic efficacies in the HD system are set perfectly from the outset of system operation and never need fine tuning or calibration [3]–[8]. However, since it is unlikely that the HD system could be so precisely-wired from birth so as to never drift, or that static HD connectivity could suffice indefinitely, a calibration mechanism is likely required for both initial wiring and tuning of the system, and ongoing re-tuning in the face of injury and age-related degeneration of system components.

The HD system has a foundational role in navigation; without it, the determination of place during motion would not be possible. Recent work has demonstrated that the HD system in infant rats is fully functional while the place and grid cell systems, which represent the animal's location in the environment, are still maturing [11]. This order of maturation is consistent with the HD system being an input to these higher centres and that it must be working before these centres can operate reliably. The directional information provided by the HD system must accurately reflect the animal's rotational motion through the world; that is, the HD system must itself be calibrated if it is to provide a faithful representation of head direction to allow the animal to accurately track its position. By definition, calibration is not possible with arbitrary, uncorrelated sensory input – the input needs to be reliably correlated with an animal's movement. It is an open question to what extent random movement (and its corresponding correlated sensory input) could suffice and if not, what kinds of movements are required to establish such reliable correlations.

We conjecture that – as with other aspects of learning – specific tasks may facilitate calibration by the repetition of experiences with reliable correlations. Infant mammals of many species including cats, badgers, and both laboratory and wild rats, exhibit characteristic ‘warm-up’ periods of stereotypical motion prior to commencing or resuming normal movements, particularly in novel environments [12]. These warm-ups, by virtue of their ubiquity and similarities across species, have been conjectured to be important for development of the underlying neural sensorimotor systems [12], although exactly in what capacity was not clear. Their use as a general preparation for sensorimotor activity is likely to serve many different neural sub-systems. We suggest that one of their outcomes is a role in calibration of the head direction system and in this study we test this proposal.

To examine how correlations in sensorimotor systems arise from behaviour and to determine their efficacy for calibration, an embodied system is required. In neuroscience, robotics platforms are increasingly being used to explore the link between physical embodiment, neural control, learning and behaviour [13], [14]. In particular, they can be used to study how physical movements can impact learning in noisy, real world environments. Real world noise is impossible to model accurately (*e.g.* as a simple Gaussian process) due to its origins in multiple ill-defined sources (motor as well as sensory), non-stationarity, potential unknown inherent biases, and possible systematic variations based on factors such as lighting conditions, temperature, time of day, proximity to noise sources and countless other environmental dependencies. For this reason, to determine if a system is robust to real-world phenomena, it is necessary to implement it in the real world. Embodied implementations have significant additional advantages: Constraints from the real world have the potential to facilitate the learning process through correlations, redundancy, and physical laws which govern how objects must move and interact, and learning processes can utilise actions that seek specific sensory experiences, modify the state of the world, or both, in a controllable and constructive manner.

Our overall aim in this paper is to show how targeted behaviour can be used to calibrate neural systems. In particular, the reported studies show how a neural HD system implemented on a mobile robot can be calibrated by a combination of neurologically-plausible learning mechanisms and the observed ‘warm-up’ behaviour in infant animals as they learn to move through the world. We chose robotics as a method of testing the performance of calibration in our HD network because it provides a way to evaluate the effects of neural mechanisms on behaviour (and behaviour on neural mechanisms) in a real world setting. For sensorimotor calibration, it is clearly important to ground actions in sensorimotor perceptions [15], particularly since we are also proposing a link between animal behaviour and HD calibration. We applied the behavioural warm-up strategies and appropriate synaptic learning rules, first in simulation and then on the real robot, and tested the resulting directional stability of the HD representation and its ability to track ongoing head movements. The studies demonstrate how specific movements, when combined with the proposed synaptic weight updates, can lead to stable and faithful representation of head direction, with performance in darkness (*i.e.* without visual cues) similar to that seen in rodents. Control studies show how the calibration breaks down if the targeted movements are not performed or if movement and sensory input are not correlated. Implications of the work are discussed with regard to how targeted motion can calibrate neural systems, the biological implementation of the proposed synaptic learning rules, calibration of robotic systems, and the potential importance of robotics in neuroscience.

### The need for calibration

For the HD activity to accurately and consistently track physical head rotations, the angular velocity of the heading representation (the *bump*) in the attractor network must reliably correspond to the angular velocity of the rat's head in the azimuth plane. During embodied motion through the physical world, the body has access to acceleration (vestibular) signals, as well as the obvious but exceptionally useful constraint that the head always returns to its initial heading after a full 360° turn. These two information sources – the vestibular motion and the heading invariant – are real-world constraints available through embodiment. In the context of the current study, we restate the HD accuracy requirement as two stability conditions which use, respectively, the two information sources, and are jointly sufficient to satisfy the requirement.

- The bump must move at a consistent speed, in both directions, throughout the entire HD network (this includes the degenerate case of there being no induced bump movement when there is no actual head movement).
- A continuous turn through 360° must return the attractor bump to its starting point.

Condition 1 ensures that the HD bump is stable, that it does not drift and that it turns evenly throughout its entire angular range. However it does not ensure that the absolute turn rate exactly corresponds to the angular velocity of the rat's head, which is realised by condition 2. A full 360° turn is a minimal condition requiring only a single landmark to afford recognition of the full turn. It provides ‘ground truth’ for the total turned angle that requires neither measurement nor calibration of any further angle tracking mechanism; alternatives to full turns, such as using angles between whiskers, total neck flexion or visual fields of view, are possible mechanisms for deriving turn angle information, but they require either prior calibration or else *a priori* knowledge of the angular extent covered by the given sensorimotor modality. While the above conditions are sufficient for satisfying the HD accuracy requirement, it is not currently possible to prove that they are necessary, since different (perhaps as-yet unknown) sensory input could lead to different learning conditions. However, the need for calibration somewhere in the system is indisputable.

In most existing models of HD networks, the synaptic efficacies in the HD system are perfectly preset so as to never need fine tuning or calibration [3]–[8]; we call this class of models the *non-adaptive attractor models*. In contrast, some computational studies have suggested that vestibular control of the HD system can be calibrated using visual input [16]–[18]. However these simulation models required that multiple visual landmarks be clearly and uniquely pre-associated with distinct head directions prior to training, and it is unclear how such precise and correct pre-associations could be effectively formed before the HD system is fully functional. In previous work we have demonstrated that pre-associations to specific head directions are not required, using a rate-coded continuous attractor network implemented on a robot [19]. Furthermore, HD drift can be eliminated and turns of equal angle in opposite directions can result in equal but opposite displacement of the HD bump (stability condition 1 above) with no reliance on visual input [20]. In the latter study we demonstrated an *adaptive attractor* model that used the turn information conveyed by a complementary class of cells, the symmetric angular head velocity (AHV) cells, to tune the HD attractor system. In so doing we proposed a novel role for these symmetric AHV cells, which previously had no known function despite their abundance in the brain. However this system did not include a mechanism for satisfying stability condition 2, to ensure that the absolute speed of the bump through the network during head turns exactly matched the speed of the head.

In the current study we extend our earlier work to include the use of a single heading landmark which does not need to be pre-associated with a given direction, and show how a 360° turn can be calibrated to return the attractor to its starting point (stability condition 2). We introduce a synaptic learning rule in a spiking neural network model of the HD system which adjusts a global turn gain parameter controlling how quickly the HD bump moves through the network for given AHV input. The gain is updated each time the HD bump position is reset by recognition of a heading landmark. Strategic movements facilitate the association of a landmark with a given head direction and elicit landmark reset events.

### Infant Mammal warm-up movements

Rat pups follow developmental milestone ‘warm-ups’ which are often carried out prior to commencing any other movements [12].

- From birth (day 0) to 3 days old (day 2), pups move about significantly with no stereotypical warm-up period.
- On days 3 and 4 most movement ceases, being limited to very small lateral head trajectories.
- On day 5 or 6 gross movements re-emerge, always beginning with small side-to-side head movements which slowly increase in amplitude, before resumption of normal motion.
- On day 6 ‘pivoting’ behaviour appears after the lateral head movements, where the rat rotates its entire body through 360° or more by walking in circles with three legs while keeping one hind leg firmly placed.
- Forward and backward ‘rocking’ motions then appear and occasionally also backwards walking.
- From day 11, warm-up sequences are shortened, some steps may be skipped, and eventually regular movements commence with no prior warm-up period.

Infant mammals of several other species display similar periods and patterns of warm-up movements during development [12]. Adult rats that have lesions of their lateral hypothalamus (resulting in a movement disorder known as lateral hypothalamic akinesia) follow a similar pattern of warm-up movements, on an accelerated timescale, when placed in novel environments during recovery from the lesion. After initially freezing in the new environment, they begin movement with small lateral head turns that rapidly increase in amplitude, followed by pivoting and then front-back rocking motions, prior to resuming normal movement [21]. When accompanied by ultrasonic vocalisations, warm-ups have been conjectured to be involved in rat pups' searching and calling for their mothers [22], however this theory does not explain why weaned adult rats with movement disorders would exhibit these behaviours. An alternative proposal [12] is that the onset, organization and similarities of these warm-up movement strategies may indicate their importance to the underlying neural systems that mediate them, and movement warm-ups may be a basic principle common to many mammalian species during times of development and re-development of these movement systems.

Because rat pups' eyes do not typically open until day 14 or 15, after the warm-up stage of their development, orienting landmarks used by pups are likely to be tactile in nature, detected by the whiskers (we used vision for the robot implementation because vision sensors for robots are cheap, common and reliable, unlike current tactile sensors and artificial whiskers which remain experimental, but the principles remain the same). Rat whiskers are extremely sensitive and possess exceptional discriminatory power. Using their whiskers, rats are able to locate and recognise objects, perceive space and navigate through complex environments [23]. Rats rarely venture far from enclosed spaces where whisking input is plentiful, but in open spaces rat whiskers have the perceptual acuity to be able to detect subtle changes in floor texture. Learned landmark-to-HD associations are hypothesized to reset the HD bump to match the known direction of the landmark [9], [24].

### Targeted movement for calibration

Calibration of sensorimotor systems in humans and other animals, also known as *sensorimotor adaptation*, has been widely investigated, usually in the contexts of visual saccades [25], [26] or arm movements [27]–[29]. Infants spend a significant amount of time touching their own faces [30] and in continuous repetition of the same movements [31]. Targeted movement is a viable strategy for learning associations and invariants in the physical world, and the recurrent effects of motion and perception; that is, correlation of action with effect, or calibration.

In robotics, calibration is a critical yet often time-consuming and expensive operation [32]. Current research efforts are focused on calibration without elaborate external sensors (self-calibration) [32], [33], in the face of only partial information [34], and through selection of specific movements and poses [35], however there are no existing practical examples of calibration of *neural* systems using behavioural strategies. Can behaviour be used to calibrate a neural system? In this study we combine all three approaches of self-calibration, unreliable real-world sensors and targeted movement to reliably and efficiently calibrate the neural HD system on a mobile robot.

## Methods

### Network Architecture

**Figure 1:**
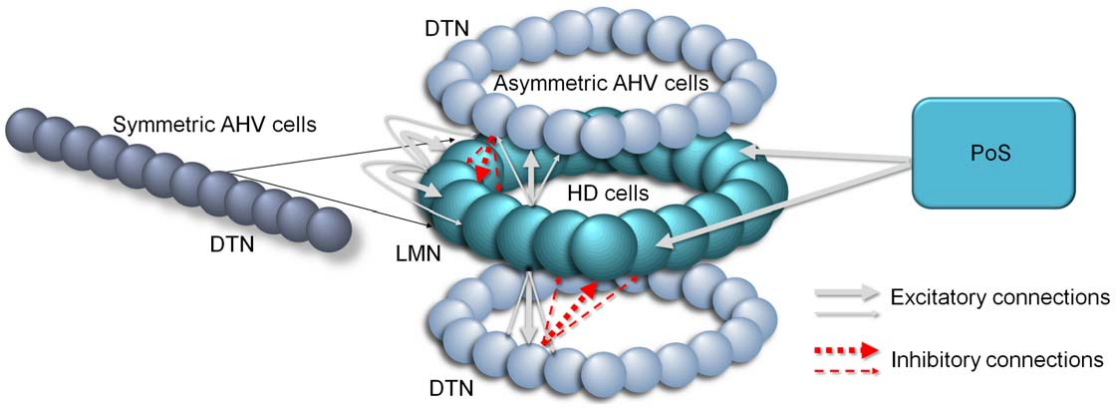
Head direction adaptive attractor network (HDAAN). HD cells excited their close neighbours strongly and more distant neighbours less strongly, and this self-excitation created the HD activity bump. Most, but not all HD system models, utilise recurrent excitatory connectivity to maintain the activity bump; those without recurrent connectivity require continuous external input to sustain the bump [3], [7]. The HD cells also excited the asymmetric AHV cells using a similar neighbourhood relation. The left turn AHV cells then projected back to the HD cells with an offset in one direction (“leftwards”) and the right turn AHV cells projected back to the HD cells with offset in the other direction (“rightwards”). These AHV projections back to the HD cells were inhibitory, and the combined effect of these two populations was to constrain growth of the bump to be within the bounds of the offset distances in both directions. The symmetric AHV cells projecting to the HD cells aided calibration for drift removal and turn equalisation (stability condition 1). PoS sent excitatory current to the HD cells to reset HD position when a landmark was recognised. DTN: Dorsal tegmental nucleus, LMN: Lateral mammillary nucleus, PoS: Postsubiculum.

The head direction adaptive attractor network (HDAAN) used in this study contained populations of four distinct types of neurons; head direction (HD) cells, left turn angular head velocity (AHV) cells, right turn AHV cells, and symmetric AHV cells (see Figure 1). Apart from the symmetric AHV cells, the network was configured similarly to non-adaptive attractor models [3]–[8], with each set of cells connected in a ring so that head direction activity “wrapped around” and restarted after a full rotation. The symmetric AHV cells were connected uniformly to the HD cell population and were not circularly arranged (see Figure 1), since they were involved in providing the calibration signal to the HDAAN, not in asymmetrical left/right movement of the HD bump. The relevant subcortical brain regions for the adaptation mechanisms hypothesised by this model are the Dorsal Tegmental Nucleus (DTN), containing the symmetric and asymmetric AHV cells [9], the Lateral Mammillary Nucleus (LMN), containing the HD cells to which the AHV cells directly project [9], and the Postsubiculum (PoS). PoS was modelled functionally, not explicitly as a group of individual neurons; the excitatory input that caused a reset of HD bump position in the network when the head faced a landmark was assumed to come from PoS, as has been commonly conjectured [9], [24].

Neurons were modelled as leaky integrate-and-fire (LIF) cells [36] and synaptic currents were modelled as fast rise, slow decay currents. The number of HD cells in the HDAAN was set to two hundred, however tests were performed to ensure results generalised to much larger numbers of cells. Synaptic connection efficacies between cells were modelled with Gaussian functions where the standard deviation controlled the width of the HD activity bump. Each HD neuron had a maximum firing rate of 150 Hz and a Gaussian-like tuning curve 110° wide, giving them firing profiles similar to HD neurons recorded in LMN [9]. Full details of the neuron and synapse models and the network architecture are available in [20].

The initial state of the system was deliberately configured to model an uncalibrated system in which correction would be required. First, the untrained HD connection weights were initialised with a circular bias, implemented as a shift in the postsynaptic target cells of recurrent excitatory HD connections. The systematic offsets in the synaptic connections caused significant drift which had to be corrected to satisfy stability condition 1. Second, the absolute speed of the bump through the HDAAN was set independently of the physical head turning speed; the turn gain therefore needed to be corrected to satisfy stability condition 2.

### Removing Drift and Equalising Turn Rates (stability condition 1)

In previous work we demonstrated that HD drift can be eliminated and turns equalised (stability condition 1) using just the information conveyed by symmetric AHV cells in the DTN [20]. For example, prior to calibration the HD bump could be rapidly drifting in one direction (at *d* deg/s) and turns of equal magnitude in opposite directions could result in different angular displacements of the bump (head turns of ±*a* deg could result in bump displacement of respectively *ga*+*d* and –*ga*+*d* deg where *g* is the turn gain). Following calibration for stability condition 1, bump drift is removed (*d* = 0 deg/s) and bump displacement is equal in opposite directions but not necessarily equal to actual head turn angle (head turns of ±*a* deg result in bump displacement of respectively ±*ga* deg). These aspects of the model [20] were retained in the current work and the equations are reproduced in this section for completeness:

At time *t*, the instantaneous firing rate of cell *i*, *r_i_*, is given by:
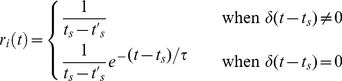
(1)where *t_s_* is the time of the last spike,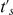 is the time of the second-last spike (hence
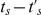
is the most recent interspike interval (ISI)), *τ* is the firing rate time constant (33 ms) and *δ* is the Dirac delta function. The instantaneous change in HD cell *i*'s firing rate is calculated as the difference between its instantaneous firing rate and its short term average firing rate:
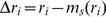
(2)where *m_s_*(.) is the short-term average, calculated as an exponential moving average with a time constant of 20 ms.

The change in the strength of the connection *w* from presynaptic HD cell *i* to postsynaptic HD cell *j* is given by:
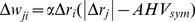
(3)where *α* is the learning rate, *r_i_* is the current firing rate of cell *i*, |*…*| denotes the absolute value of a term and *AHV_sym_* is the current input from the symmetric AHV cells. *AHV_sym_* input was assumed to be rate coded, with firing rate (and hence input current to the HD neurons) proportional to the head turning speed. All HD neurons received the same *AHV_sym_* input. For this reason, the *AHV_sym_* neurons were modelled as just one neuron that had connections to every HD cell. In biological systems, such a large connectivity fan-out is not usually possible; however this simplification in our model system reflected our view that each real HD neuron receives identical *AHV_sym_* input, limited only by fan-out constraints that require a larger number of *AHV_sym_* neurons in practice.

Intuitively, the calibration works as follows. If the bump is moving too quickly, then *AHV_sym_* is less than the absolute change in the HD neuron's firing rate, so the term |Δ*r_j_*| – *AHV_sym_* is positive. Connections are weakened from those presynaptic HD cells whose firing rates are decreasing (Δ*r_i_* is negative), and strengthened from those presynaptic HD cells whose firing rates are increasing. HD cells with decreasing firing rates are necessarily on the trailing edge of the bump, while cells with increasing firing rates are on the leading edge. Weakening the weights from cells on the trailing edge (where the bump just came from) to the cells which are currently firing retards the movement of the bump because it reduces the impetus of the bump to move forward. Similarly, strengthening the weights from cells on the leading edge (where the bump is moving to), has the same effect because it is trying to stabilise the bump back in its current position. Hence when the bump is moving too quickly, the net effect of the weight updates is to decrease its speed. Conversely, when the bump is moving too slowly, the net effect of the weight updates is to speed it up. For full details of how stability condition 1 was satisfied and results of experiments see [20].

The learning rule for condition 1 was modified in two ways to improve performance for this study. Firstly, autapses (excitatory connections from a cell to itself) to HD cells were omitted. HD autapses are not functionally useful for control of movement of the HD bump, since they excite only themselves and therefore cannot assist in propagation of the peak of HD activity to surrounding cells. Removal of these connections resulted in faster convergence to ideal synaptic efficacies in the HD connections. Secondly, the HD connection efficacy decay used in the earlier study was replaced with an explicit presynaptic sharing where efficacies were shifted between adjacent connections, from stronger to weaker synapses. Each postsynaptic HD cell *j* shared connection weights from its presynaptic cells *i* and the circularly adjacent presynaptic cells *i*+1 and *i*−1. The change in the weight from cell *i* to cell *j* was given by:
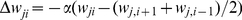
(4)where *α* is the learning rate. Recent evidence indicates that synaptic resources are shared between close synapses in neurons [37]. In the current study, sharing resulted in a smoothing of any noise in the synaptic connections which, in conjunction with the ongoing calibration, resulted in connection weights that tended towards their ideal Gaussian distributions. These two modifications to the drift calibration resulted in superior convergence and HD tracking performance.

Note that the calibration performed for condition 1 eliminated drift and ensured that the *relative* turn rates in both directions were equal, but it could not ensure that the *absolute* turn rate would track actual head position in the world, since the absolute turn gain *λ* remained uncalibrated. Calibration of the turn gain is the focus of section 2.3.

### Turn Gain Calibration Mechanism (stability condition 2)

**Figure 2:**
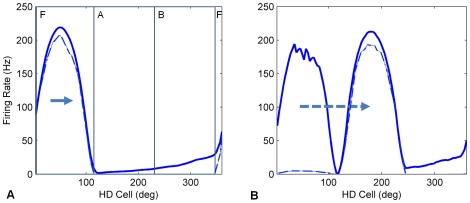
Turn gain is updated when the HD bump is reset by landmark input. (A) The bump is travelling to the right, as indicated by the arrow, at approximately 40 deg/sec. However the head is turning at approximately 65 deg/sec, meaning that the HD turn gain is too low. The dot-dash line shows the instantaneous firing rate; that is, the HD cells which are currently firing, denoted as region F. These cells do not send a gain adjustment signal if they receive landmark input. The solid line shows the firing rate trace; the region of the network immediately behind the bump, denoted as region B, shows higher trace activation than the region just ahead of the bump, region A. HD cells in region B send a gain reduction signal if they receive landmark input while cells in region A send a gain enhancement (discussion of how this signal may be implemented in nervous systems is in section 4). The demarcation between regions A and B is based on the firing rate trace activation threshold of 10 Hz. (B) 120 ms later, the HDAAN has received landmark input causing the bump to jump ahead to the landmark location at 180°. Since most of the HD cells which received landmark input were in the low activity-trace region (region A), the turn gain is increased overall.

We modeled the calibration of HD turn rate to real world turn rate by adjusting a global gain parameter, *g*, on the vestibular input from the asymmetric AHV cells, *AHV_asym_*, to the HDAAN (*i.e.* total turn input to the HD network is *g.AHV_asym_*). If the HD bump was moving too slowly, the gain was increased to amplify the effect of vestibular input on the bump, which sped the bump up. Conversely, if the bump was moving too quickly, the gain was decreased. A single gain parameter *g* controlled the overall efficacy of the vestibular input to the HDAAN and hence the overall speed of the bump during head turns. The gain parameter was set by a local learning rule which operated at the level of individual HD cells. Each HD cell preserved a memory of its recent firing history by maintaining a decaying trace of its highest recent firing rate. HD cells could also receive input current from PoS when the head was facing a recognized landmark. Landmark recognition and its association with specific head directions in the HD network was assumed to be performed by the PoS (see section 2.5 for details). If landmark association input was received by an HD cell which was not currently firing, then the need for landmark reset of the bump position indicated that the bump had not been correctly tracking real head direction and the gain needed to be modified. Unlike previous studies, only a single landmark was required and it could be positioned at any arbitrary angle from the head. When gain adjustment was needed, its increase or decrease was controlled by the recent firing history of the cell. If the cell had been firing recently, indicated by a firing rate trace above a certain threshold (set to 10 Hz), then the bump was being reset backwards to a position it recently occupied, in which case it had turned too quickly and the gain needed to be reduced; the individual HD cell sent a signal which very slightly reduced the gain. Conversely, if the cell hadn't recently been firing, indicated by a firing rate trace below the threshold, then the bump was being reset forwards to a position it hadn't recently occupied, in which case it had turned too slowly and the gain needed to be increased (see Figure 2).

Turn gain calibration is implemented as follows:

The firing rate trace of HD cell *i* is calculated exactly as for Eqn 3 above, except that the time constant *τ* is increased to give a longer memory of recent firing (*τ* = 2000 ms).

When actual head direction approaches a landmark heading, and landmark reset input impinges on the HDAAN, the 360° calibration learning is activated; that is, calibration requires landmark reset input from PoS in order to provide output signals to adjust the turn gain in the HD network. HD cells that are currently firing, as indicated by an instantaneous firing rate above 1 Hz, also do not adjust the turn gain, since cells that are already firing when landmark input is received are already representing head direction accurately. However, if a cell *i* is receiving landmark input and the cell is not currently firing, but has recently been firing as indicated by a firing rate trace activation of above 10 Hz, then the activity bump has already moved past the location where it should be, so the cell sends a gain reduction signal, *g_r_*:
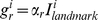
(5)where *α_r_* is the learning rate for gain reduction and *I^i^_landmark_* is the landmark input current to the HD cell. Conversely, if a cell *j* is receiving landmark input, is not currently firing, and has not recently been firing as indicated by a firing rate trace activation of below 10 Hz, then the activity bump has not reached the location where it should be, so the cell sends a gain enhancement signal, *g_e_*:
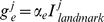
(6)where *α_e_* is the learning rate for gain enhancement. The learning rate for gain enhancement, *α_e_*, is set to 1×10^−6^, while the learning rate for gain reduction, *α_r_*, is set to 1.5*α_e_*, to compensate for the slightly delayed fall in instantaneous firing rate after the bump has passed a cell (the delay reduces the number of cells sending gain reduction signals). The total gain adjustment signal, Δ*g*, is the sum of the individual gain adjustments:
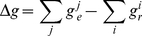
(7)


As soon as an HD cell commences firing (*i.e.* if the HD bump is reset to the landmark position), it ceases sending gain adjustment signals since when it is firing it meets the criteria for the HD bump being correctly positioned, meaning that no gain adjustment from that particular HD cell is appropriate.

### Learning Suppression and Annealing

When an HD reset which actually moves the HD bump occurs, calibration for stability condition 1 is suppressed for 1 second. This suppression is necessary because the movement of the bump to a new position clearly induces large HD activity changes, interpreted by the stability calibration mechanism as instabilities in the network and causing large maladaptive updates to the HD connections. By suppressing connection updates for a short time, the time-weighted average HD activity, which is used by this calibration mechanism to eliminate drift and equalise turn rates in the HDAAN, is allowed to re-settle to the new HD bump position, after which no maladaptive connection updates occur.

The initial learning rate is set to 20 times the base learning rate. At the end of each second of training, the learning rate is reduced by 0.5%. This reduction means that for each minute of training the learning rate falls by about 26% (1–0.995^60^) and in just under 10 minutes falls back to its original baseline value, after which no further reduction occurs. Annealing the learning rate in this way dramatically shortens the training time for the network, since it allows large adjustments to the connection weights to be made initially while still allowing very fine adjustments to be made when the weights have approached optimal calibration.

### Landmark Resetting of HD Position

**Figure 3:**
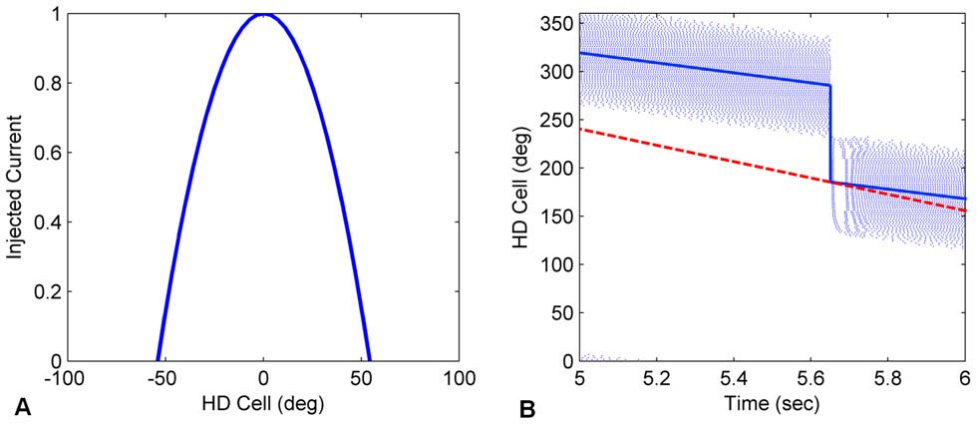
Landmark reset. (A) The activity template injected into the HDAAN when a landmark is recognised. The template shows the maximum current injected when the head is facing the landmark exactly; injected current falls to zero when the head deviates from the landmark by more than ±3 degrees. (B) The HD bump position being updated by the landmark input during a head turn. Each dot represents a spike of an HD neuron. The solid blue line tracks the centre of the HD activity bump, which corresponds to the head direction represented by the network. The dashed red line tracks the actual head angle of the head. At time 5.65 secs the head reaches the landmark at 180° but the HD network is trailing behind (turning too slowly). The injected landmark reset current resets the head direction representation to the landmark position, from where the turn continues.

Landmark recognition and the association of a landmark with specific head directions in the HD network are assumed to be performed by the PoS. Experimental studies [38], [39] and other models (*e.g.* [3]) have investigated this phenomenon in detail, including the dependence of HD representation on landmark direction, relative HD orientation in different environments and response latency to abruptly reoriented landmarks. These issues are not the focus of the current study. In the current model, the important characteristic of a landmark reset is that it causes a discontinuous jump in HD bump position to a prior known head direction irrespective of how that association was made or the exact reset mechanism. When actual head direction is aligned with a landmark, input current, *I_landmark_*, is injected into the HDAAN at the bump position for that landmark. Maximum current is injected into the cell at the centre of the landmark location and the current tapers off to the cells on either side (see Figure 3a) according to:
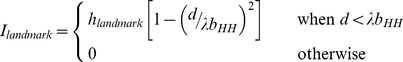
(8)where *d* is the absolute distance, in number of cells, from the centre of the landmark location, *b_HH_* defines the width of the excitatory HD connections which create the activity bump (see Figure 1), and *λ* is a parameter based on the relative widths of the activity bump and the excitatory HD connections (the activity bump width is greater than the connection width from each neuron, since surrounding neurons also project to neurons further away, extending the bump; *λ* was set to 1.5 in all simulations). Additionally, the injected current attenuates rapidly when the head is not facing directly towards the landmark according to the landmark heading factor *h_landmark_*:
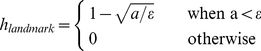
(9)where *a* is the absolute angular difference in degrees between the current real head direction and the landmark position, and *ε* is the tolerance (for these studies *ε* was set to 3 degrees). A small landmark input current therefore begins being injected into the HD cells when the real head direction is within 3 degrees of the landmark, then reaches a maximum when the head direction matches the landmark exactly, then falls off equally as rapidly as the head continues to turn (see Figure 3b). If the head stops while facing the landmark, current is injected continuously into the HDAAN.

### Pioneer Robot Implementation

**Figure 4:**
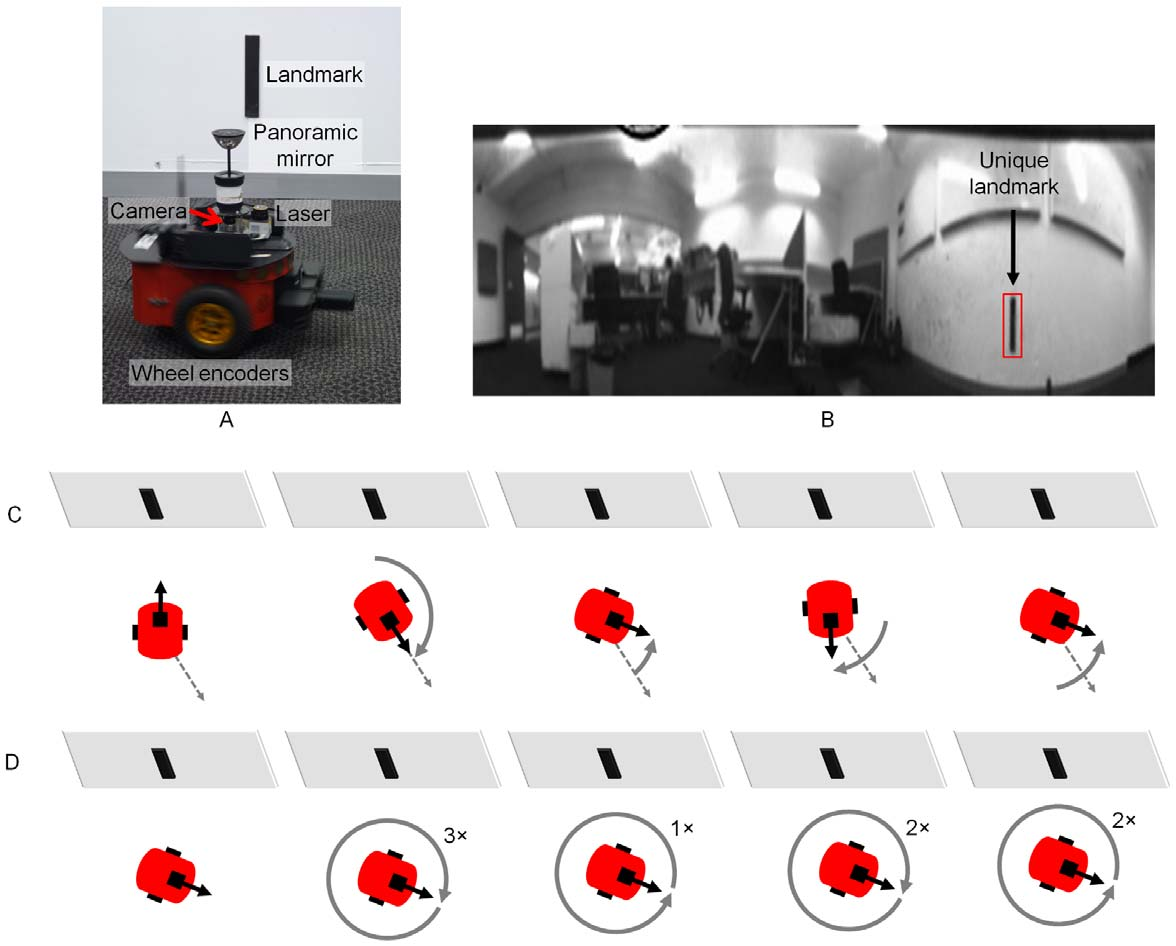
The robot platform, testing environment, and movement schemes. (A) The robot was equipped with a panoramic imaging system, laser and wheel encoders, which provided self-motion data and landmark tracking capabilities. The environment consisted of a typical office room with a distinct and unique black landmark against a white wall background. (B) Panoramic image from the robot's camera showing the testing environment and the landmark. (C) Movement scheme for the small head turn routine, consisting of small alternating turns, centered around a random initial orientation (shown by the dashed arrow). (D) Movement scheme for 360° calibration, consisting of alternating turns of 1 to 3 revolutions in either direction.

Experiments were performed on a Pioneer 3-DX MobileRobots robot (Figure 4a, www.mobilerobots.com/ResearchRobots/ResearchRobots/PioneerP3DX.aspx). The testing environment was a standard office room. A unique black landmark cue was located against a white wall, as shown in Figure 4a and b. The egocentric bearing to the landmark was calculated using intensity profile vision processing techniques [40]. Detection of the landmark was not perfect, meaning that landmark direction used for calibration of the HDAAN was noisy.

The robot was commanded to follow an alternating sequence of small lateral head turns and 360° head calibration routines, switching routines every minute. Small head turns consisted of the robot turning to a random absolute orientation, and then commencing a period of small alternating turns of 40° to 80°, centered on that absolute orientation (Figure 4c). 360° calibration consisted of the robot performing alternating turns of 1 to 3 full revolutions in each direction (Figure 4d). Command rotational velocities for both routines varied randomly between 25 and 100 degrees per second. Sensed rotational velocities, akin to the vestibular sense in mammals and used as the rotational input to the HDAAN, came from the wheel velocity encoders which, on the robot used for HD training, systematically under-represented total turn angle by approximately 8% in addition to being noisy, and so accumulated major error over time. Calibration of the HD system needed to be robust to these errors.

## Results

Results of the HDAAN trained on simulated head direction movements are presented first, followed by training based on visual flow from a mobile robot platform.

### Simulation – 360° turn gain calibration only

**Figure 5:**
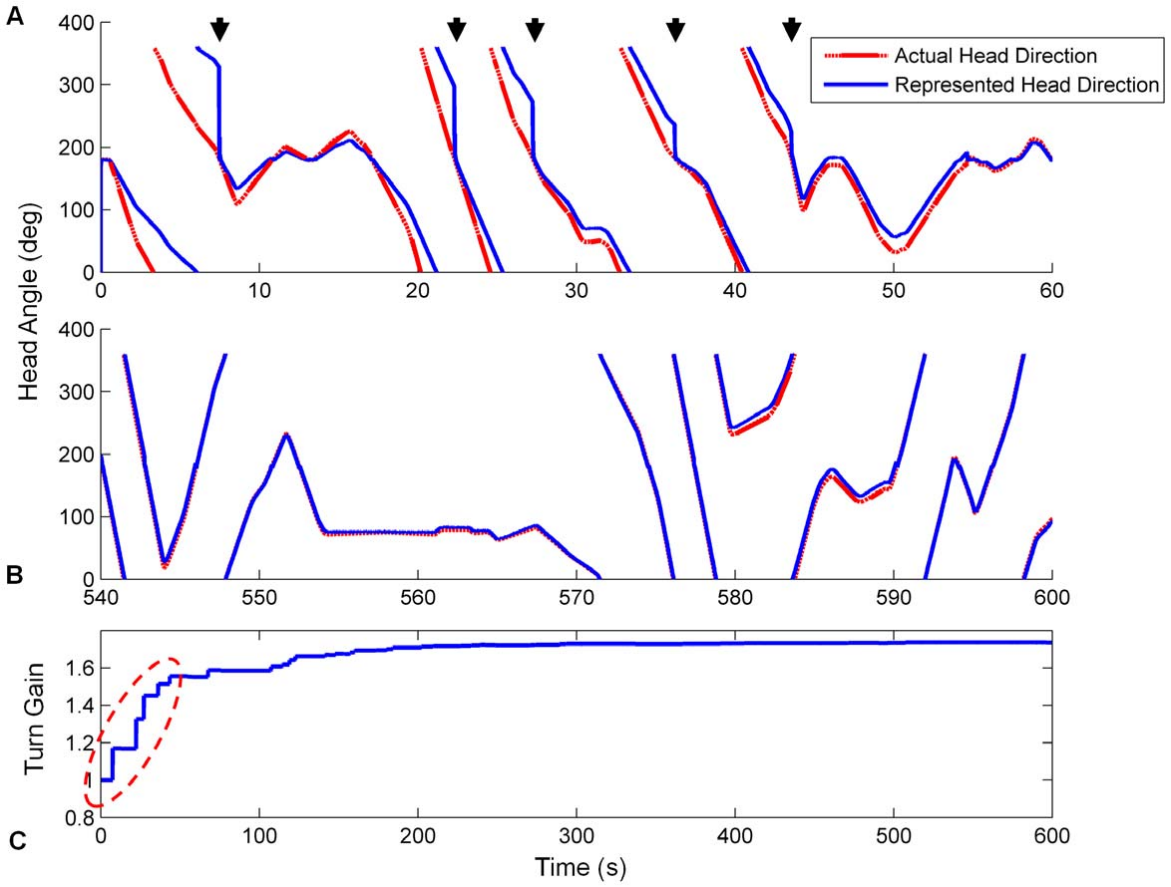
Head direction tracking improved as turn gain was calibrated. Actual and represented head directions were tracked for the first and final 60 seconds of training (respectively graphs (A) and (B)). X axis – time in seconds, Y axis – head orientation in degrees, dashed red – actual head position, solid blue – head position as represented in the HDAAN. (A) At the commencement of training, the HD bump consistently under-turned and therefore lagged behind actual head direction. At this stage, large HD landmark resets occurred each time the actual head direction passed through the landmark position at 180° (indicated by solid vertical lines in the represented head direction and marked with arrows). The HD resets became progressively smaller as training progressed due to adjustment of the turn gain by the ongoing calibration. (B) At the completion of training, the represented head direction tracked actual head direction almost perfectly. The slight discrepancies apparent between actual head direction and the HD bump position from 580 to 590 seconds were due to HD reset being performed during fast head turns; during the reset, which took a short time, the head continued to turn slightly past the reset position, causing head direction to lag slightly even when the turn gain was correctly calibrated. (C) Turn gain was initialized to 1.0 prior to training and within 5 minutes (300 seconds) it reached its correctly calibrated value of 1.735, from which time the calibration remained stable. The first five gain adjustments (circled in (C)) correspond to the five arrowed head direction resets shown in (A).

To demonstrate the functioning of 360° turn gain calibration for stability condition 2 in isolation, results are first presented for an HDAAN with no bias in the connection weights so that tuning for stability condition 1 is not required. Random turns in the range −90 to 90 degrees/s were initiated for random durations of 0 to 15 s. At random times (on average once every second), the turn rate was randomly changed by adding a value in the range −45 to 45 degrees/s. Turns were allowed to reverse direction, however absolute turn rate was capped at 135 degrees/s. Whenever a new turn was to be initiated, a similar period of rest (*i.e.* a turn at 0 degrees/s) could instead occur with 10% probability. The network was deliberately initialised with an incorrect low turn gain, causing the HD bump to move too slowly. A simulated landmark at 180° reset the HD bump to the correct position whenever the head crossed that heading. Training occurred for 600 s (10 minutes) of simulated time. The training successfully increased the turn gain and allowed the HDAAN to reliably track head direction (see Figure 5 for results from a typical 10 minute training run).

### Robot

#### 360° turn gain calibration

**Figure 6:**
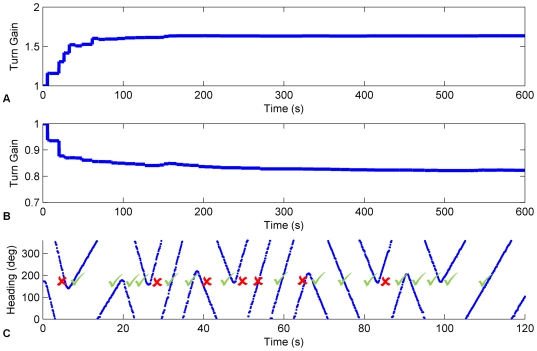
Turn gain converged rapidly for turns on the mobile robot platform. Convergence occurred despite unreliability of the visual tracking of the landmark that was used for calibration. Turn gain was increased when it started too low (A) and decreased when it started too high (B) as needed to reliably track robot heading. The bottom panel (C) shows the robot's perceived heading to the landmark at each time point that the landmark was detected, during the first two minutes of training. Imperfections in the robot's visual tracking of the landmark caused approximately 30% of the landmark alignments with 180° (which should cause landmark reset of the HD bump) to be neglected in the first two minutes (red crosses in (C)). Despite the variable landmark tracking success, turn gain was quickly optimised, indicating robustness of the training.

In a manner similar to the simulation results presented above, we first demonstrate the functioning of 360° turn gain calibration for stability condition 2 in isolation, using turn data gathered from a Pioneer mobile robot platform (see Methods for details of data collection from the robot). The robot was instructed to turn on the spot from one to three full turns in alternating directions at random speeds between 14 and 70 deg/s. The robot also visually tracked a landmark fixed to an adjacent wall, and when the robot turned to directly face the landmark, the HD bump position was reset to 180° (the bearing associated with the landmark). Two HDAANs were tested with the same 10 minute-long robot turn sequence, one with an initially low turn gain that caused the activity bump to move too slowly, and one with an initially high gain where the bump moved too quickly. In both cases the training successfully adjusted the turn gain (see Figure 6a and b) and allowed the network to reliably track the robot heading.

The robot's visual tracking of the landmark was not perfect, resulting in gaps and jumps in the perceived heading of the landmark from the robot (see Figure 6c). These heading jumps caused the perceived landmark heading to skip the 180°±3° region needed to cause a landmark reset approximately 30% of the time; despite this, the turn gain converged rapidly to its optimal value, which illustrates the robustness of the learning algorithm.

#### All calibrations

We next performed simultaneous bump drift correction, turn equalisation and 360° gain calibration on the mobile robot for an HDAAN with biased HD connection efficacies. The systematic bias in the HD connections caused the bump to drift in one turn direction continuously, completing a full 360° rotation in just four seconds (see [20] for details). To follow a similar (although accelerated) developmental timeline and similar warm-up strategies to infant rats, the robot's motion profile proceeded in two stages as follows:

- A 15 minute period of lateral head rotations of small amplitude, corresponding to rat pup behaviour at days 5 and 6, alternating with 360° rotations, which appear in rat pups shortly afterwards on day 6. Each period of behaviour, either small lateral head rotations or 360° rotations, lasted for 60 seconds duration before switching to the alternate behaviour. After 60 seconds of 360° rotations, the following 60 seconds of small head rotations occurred around a random heading that was fixed for this 60 second period but changed for the next (corresponding to a given random body position for the rat). For the full 10 minutes of this alternating behaviour, calibration for stability condition 1 only was functioning. For the robot, this stage of HD calibration is used to ensure that turns of equal speed in opposite directions result in equal speed displacement of the HD bump.
- A final 15 minute period during which alternating behaviour continued but with all calibration mechanisms active simultaneously. Operation of the calibration mechanisms at this stage worked under the assumption that the HD bump was now relatively stable and drift-free due to training in stage 1. A stable HD bump meant that a landmark position could be reliably associated with a given heading representation in the HDAAN, allowing turn gain calibration to commence. The heading at the commencement of each 60 second small rotation period became the landmark heading for the following period of 360° rotations (corresponding to a landmark that we conjecture is learned by the rat during its small head rotations). For the robot, this final stage was used to calibrate the absolute turn gain (stability condition 2).

**Figure 7:**
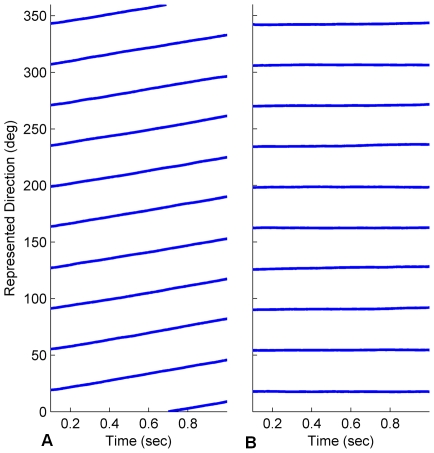
Head direction stability was greatly improved by training. The graphs track the centre of multiple HD bumps started at one of many positions in the HDAAN. (A) Prior to training, a head direction bump initiated at any one of 10 positions around the HDAAN drifted continuously. (B) Following training, drift was eliminated and the bump was stable in all positions.

**Figure 8:**
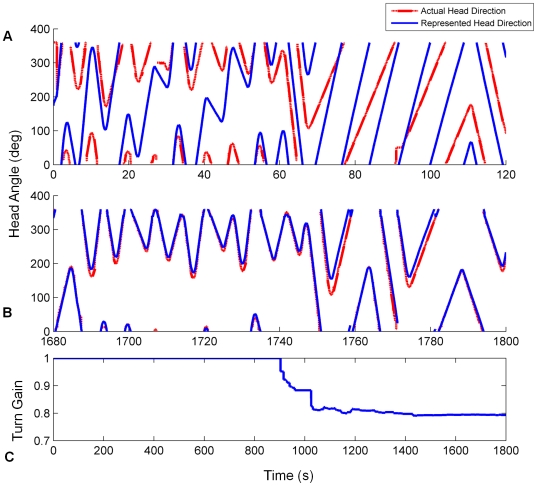
Head direction tracking improves as drift and turn gain are calibrated on the mobile robot. Actual and represented head directions are tracked for the first and final 120 seconds of training (respectively graphs (A) and (B)). X axis – time, Y axis – head orientation, dashed red – actual head position, solid blue – head position as represented in the HDAAN. (A) At the commencement of training, represented head direction drifts continuously upwards resulting in poor tracking of actual head direction. (B) At the completion of training, the represented head direction tracks actual head direction almost perfectly after the elimination of drift and equalization of turn rates (stability condition 1) and turn gain calibration (stability condition 2). (C) Turn gain is initialized to 1.0 prior to training and after 15 mins (900 secs) turn gain calibration commences. In the final 5 minutes of training the gain is stabilized at its final value.

The systematic bias in the initial HD connections caused the bump to drift in one turn direction continuously (see Figure 7a) and exhibit poor tracking of real head direction (see Figure 8a). After training, the systematic bias was removed, ensuring that the bump was stable in all positions in the HDAAN (see Figure 7b), and the turn gain was calibrated to reliably track real world head direction (see Figure 8b and c).

### Performance in darkness

**Figure 9:**
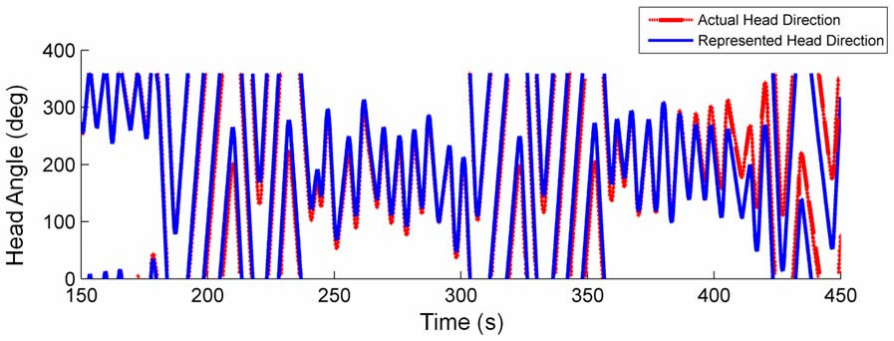
Typical head tracking in darkness maintains reasonable accuracy for several minutes. Actual and represented head directions are initially aligned at 150 s; over the next 5 minutes (300 s), angular disparity slowly increases. The error arises due to slight residual imbalances (directional biases) in the HD connections, causing the HD bump to move faster in one direction than the other in certain bump locations, as well as a small speed-dependent (nonlinear) response of the HD bump to varying input current from the asymmetric AHV cells.

The HDAAN, fully trained for bump drift correction, turn equalisation and 360° gain calibration on the mobile robot platform as in the previous section, was presented with turn data in simulated darkness – that is, turns were performed with no visible landmarks, such that reset of the HD bump position was not possible. During continuous turns with alternating one-minute periods of small lateral rotations and 360° turns, the HDAAN tracked actual head direction closely for several minutes before a large angular disparity became evident (see Figure 9). Smaller amounts of angular drift has been observed in the HD system of blindfolded rats in a similar amount of time [41], however the rats' behaviours and, importantly, total turn angles while blindfolded, were not reported in that study. In general, rats would turn sporadically, not continuously, and would be unlikely to make as many full 360° turns (20) as made by the robot in the 5 minutes shown in Figure 9.

### Importance of movement strategy for calibration

To illustrate the dependence of correct calibration on movement strategy, movements were modified to test whether calibration would be successful. Three movement modifications were examined.

- Small lateral head turns were performed as normal, but during the time when 360° turns would normally be conducted, the head was held still.
- 360° turns were performed as normal, but during the time when small head turns would normally be conducted, the head was held still.
- Sensory input was uncorrelated with head motion.

**Figure 10:**
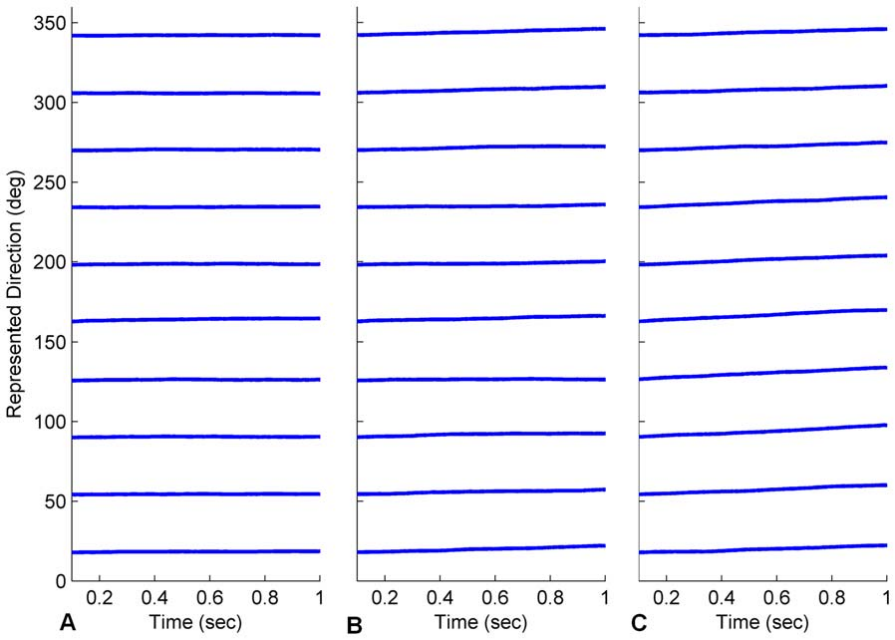
Bump stability varied with different modifications to movement strategy. (A) Small lateral head turns, but no 360° turns. (B) 360° turns, but no small head turns. (C) Sensory input uncorrelated with head motion. See text for discussion.

**Figure 11:**
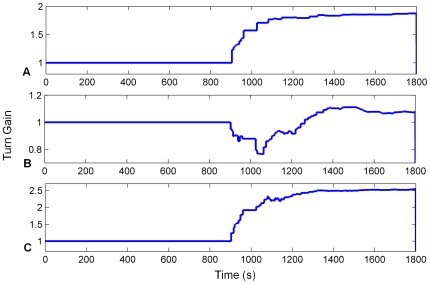
Turn gain calibration varied with different modifications to movement strategy. In all cases turn gain should have stabilised around 0.8 (see Figure 8C). (A) Small lateral head turns, but no 360° turns. (B) 360° turns, but no small head turns. (C) Sensory input uncorrelated with head motion.

Calibration failed in all cases (see Figures 10 and 11) and the failures occurred in systematic ways for the different movement modifications, indicating specific effects of movement type on calibration outcome. When small head turns alone were performed (case (a)), stability of the HD bump was excellent, with no drift apparent from any bump starting position (see Figure 10a), indicating the importance of these small head turns for satisfying stability condition 1. However the lack of 360° turns meant that turn gain calibration was not possible (see Figure 11a); in fact, with the small head turns alone, head direction resets almost always occurred from alternating directions, causing the sign of the gain adjustment to usually be erroneous, resulting in an overall gain increase instead of the desired decrease (compare Figure 11a with Figure 8c). When 360° turns alone were performed (case (b)), stability of the HD bump was compromised (see Figure 10b), with some variable amounts of residual drift remaining throughout the network. Because of the residual drift causing continuous bump position changes, gain calibration was not possible even with the execution of 360° turns, and gain varied widely throughout the training (see Figure 11b). When sensory input was uncorrelated with head motion (case (c)), bump stability was further compromised (see Figure 10c) since HD synaptic weight changes were almost as likely to increase HDAAN bias as decrease it. Because of the lack of correlation between head direction and HD bump position, HD resets to landmark locations usually appeared as forward jumps (since, with all the HD positions the bump could have recently occupied, it was unlikely to have recently been at the landmark position). Consequently gain was usually increased each time landmark reset occurred, resulting in a large erroneous final gain value (see Figure 11c).

## Discussion

### Targeted embodied movement facilitates calibration of neural systems

We have shown how exact, static pre-wiring of the HD system is not required for accurate orientation tracking. Instead, targeted movement strategies, combined with appropriate synaptic learning rules, can be used to calibrate a model of the neural HD system on a mobile robot platform, allowing the robot to reliably track real-world head direction. Successful calibration is dependent on execution of the required movement strategy; if specific movements are removed from the action repertoire, calibration fails in systematic ways. The movement must also be correlated with sensory input, highlighting the importance of embedding the body in the environment so that actions consistently impact on perceptions. Real-world constraints, such as the physical invariant that a full 360° rotation always results in return to the original heading, can greatly simplify the learning problem.

Several considerations motivated implementation of the HD calibration on a mobile robot. First, it demonstrated that the hypothesised calibration mechanism is robust to real-world noise. The robotic version of the vestibular sense, coming from the wheel rotation sensors, was biased and noisy, as was the visual determination of landmark direction. Sources of real-world noise are often unknown and the noise distributions are typically uncharacterised. Simulations of procedures and adaptive algorithms using simulated injected noise are therefore inadequate for determining robustness to noise in the real world. On the particular robot used for the HD calibration tests, turn angles were systematically under-represented by approximately 8%, and approximately 30% of landmark alignments with the robot's heading were missed, yet calibration successfully completed.

Second, implementation on a robot allowed for grounding of the required movements in the real world [15]. Similar to the distinction between simulated and real-world noise, the presumed effects of action on perception may vary significantly in the real world due to sensor placement on the robot, less-than-ideal response of sensors to different materials (*e.g.* acoustic sensors to soft walls; optical sensors to reflective or shiny surfaces) and intrinsic sensor and motor variability. To truly test our hypothesis that targeted movement can calibrate neural systems, real world implementation was imperative.

Third, recognising that calibration continues to be a significant challenge to those in the robotics community [32], we suggest that this study further demonstrates how biology can afford practical solutions to engineering problems. This study is the first to show how a neural robotic system for head direction can self-calibrate with noisy (and sometimes absent) inputs using targeted movements. This result is particularly relevant to brain-based robot navigation systems such as RatSLAM [19], [42], which has achieved several seminal navigation and mapping results [40], [43]. RatSLAM, like more traditional probabilistic robot navigation systems, is not widely deployable without either pre-training or parameter tuning. Its neural basis in place and grid cells makes it particularly suited to the calibration methods presented in this paper, affording it a means of autonomous self-calibration that is not available to other mapping methodologies.

More generally, the study shows how neural systems can be calibrated using embodied movement strategies. Self-calibration [32], [33], in the face of partial information [34], and with specific pose selection [35], are currently salient topics in robotics research. Mechanical components wear and accrue damage over time, which can be particularly significant for mobile robots that are deployed long term such as in factory and warehouse delivery operations. In a future study, we plan to test a calibration algorithm on our robot where the need for HD recalibration is indicated by the magnitude of the head direction resets which occur. When these exceed a threshold, the robot can re-initiate calibration behaviour; additionally, the calibration learning rate and annealing schedule can be set relative to the magnitude of the head tracking error.

Finally, there is growing acceptance of the importance and effectiveness of robotics in the modelling of biology, particularly in the neurosciences. Biological processes are best modelled in the context of real-world constraints and contingencies [13] in order to discover the general principles underlying their function [14]. These principles are often concealed under the colossal detail and complexity inherent in most biological systems, and many times can only be revealed by making simplifications and abstractions from this reality. The danger of making these abstractions is in compromising a model's relevance to real-world phenomena. Robotics can mitigate this risk, by re-introducing and testing the real-world relevance of the abstracted principles.

We propose that the stereotypical developmental warm-up movements, undertaken by infants of many mammalian species and adult animals with certain movement disorders, may be used for calibration of their neural head direction systems. The small-amplitude head turns which begin on day 5 or 6 serve two purposes. Firstly, they facilitate calibration of bump movement such that equal-speed turns in opposite directions cause equal-speed movement of the bump (stability condition 1). Secondly, they allow the animal to learn a landmark directly in front of it and to associate the landmark with a specific head direction represented in the HD network. With the formation of this stable association between a landmark and a given head direction, the animal can then detect when it has turned a full circle through recognition of the landmark when it completes a full 360° rotation. The 360° movements have a special purpose within the proposed calibration mechanism, since a 360° turn ‘grounds’ the calibration within a physical invariant and allows calibration to function from a single learned landmark. The 360° turns are used for setting the overall gain of the bump movement (stability condition 2). These movements rely on stability condition 1 already being met, so appear in animals subsequent to the small lateral head turns. To test the hypothesis that warm-up movements are used for calibration of the HD system, calibration should be disruptable by immersing rat pups in an environment with constantly rotating tactile cues. Older rats can be tested with rotating visual cues as we have previously suggested [20]. Behavioural indications that the HD system is functioning improperly would be a bias in the rat's turn choices during path integration with no external cues; the bias should be in the same direction as the earlier applied continuous environmental rotation. Alternatively, neural recordings of the HD system should be able to reveal instability or drift in the HD bump directly.

### Comparison with other HD models

We have previously shown in the head direction adaptive attractor network (HDAAN) that calibration to remove HD bump drift and equalise turn rates in both directions does not require visual input [20], relying instead on input from symmetric angular head velocity (AHV) cells in the dorsal tegmental nucleus (DTN), which have been hypothesised to be driven by the vestibular and motor efference systems. Calibration of the absolute turn gain to ensure that a 360° turn results in exactly one rotation of the HD bump through the HDAAN, as presented in the current study, requires just one landmark to be associated with the HD network and the association can be at any arbitrary position.

The majority of models of the HD system – those we have deemed the non-adaptive attractor models [3]–[8] – assume that the HD system does not require calibration. Even those models [3], robotics applications [44] and experimental studies [38], [39] which specifically address resetting of the HD bump position by learned landmark locations assume that, once the absolute head direction is reset by a learned landmark, subsequent head turns are tracked reliably by the HD system with no further calibration requirements. The reliable tracking presumably occurs by vestibular input where the gain has already been manually preset.

The few models that have previously addressed the issue of HD calibration have all required many visual landmarks [16]–[18]. More importantly, all prior studies have assumed that the landmarks are already uniquely and accurately associated with their correct HD bump positions within the HD network prior to HD calibration; the role of calibration in these studies was limited to learning how to move the bump with vestibular input while using landmark input as the training signal, so that the bump position could be updated in darkness, for example. However, it is unclear how these correct and accurate HD associations with landmark positions could be made before the network learned to update the bump position based on vestibular input (*i.e.* prior to calibration). While a single landmark could be learned at an arbitrary position of the HD bump, as in the current study, an unposited mechanism would be required to update the HD bump when the head rotated to the next landmark, so that the bump could be in the correct position to learn the next association. This is a chicken-and-egg dilemma, in effect requiring a fully functional HD network prior to calibration. The HDAAN, with its *adaptive attractor* model of the HD system, solves this dilemma by requiring just one landmark which can be learned at any arbitrary HD bump position.

Whilst the connection weight update algorithm hypothesised in this study operates on the recurrent excitatory connectivity between the HD neurons, the principle underlying its operation is general and is applicable to any HD model with connections between neurons of differing preferred directions. The hypothesised calibration mechanisms are therefore not reliant on recurrent excitation and will function equivalently if added to non-recurrent models. In the case of such models [3], [7], the connection weight update would need to be applied to the offset inhibitory connections to the HD neurons (*i.e.* the connections from the asymmetric AHV cells), with the signs of the weight updates negated to compensate for their inhibitory, rather than excitatory effect on the HD neurons.

### Stability of the learning rules

The size of the HD bump in the HDAAN is both set by and limited by the offset inhibitory connections from the asymmetric AHV cells, not by the strengths of the recurrent HD attractor connections. Therefore changes in the strengths of the HD connections, within reasonable limits, do not result in significant changes to the HD bump extents (maximal firing rates within the bump may be affected however). For this reason, and due to the Hebbian-like nature of the HD connection weight update rule which ensures that synaptic connections between any two HD neurons are updated only if both neurons are already firing (see Eqn 1), the HD bump will not fade, split or otherwise dissipate through the network during training. The ability of the HDAAN to maintain a single activity bump of stable size is therefore not compromised for a broad range of starting conditions, excitatory and inhibitory synaptic efficacies and learning rates.

### Biological plausibility

The 360° turn gain calibration is implemented in the model through a mechanism using local inputs to modulate an increase or decrease in the strength of the turn signal to the HD cells. The gain is updated each time the HD bump position is reset by recognition of a heading landmark. Each HD cell adjusts the gain based solely on its own firing history when it receives landmark input. The inputs to the gain calibration mechanism therefore require nothing more than local knowledge, at each HD cell, of its instantaneous and average firing rates, and its current sources of input (specifically, each HD cell responds with a calibration signal when it receives landmark reset input from the postsubiculum (PoS), as long as that HD cell itself is not currently firing).

Average firing rate, or a memory of recent firing, as calculated in Eqn 3, may be maintained neurophysiologically by intracellular calcium levels. Whilst these have not been measured for HD cells specifically, intracellular calcium transients, induced by spiking in cortical pyramidal cells, decay with time constants in the range of 1.5 to 3.0 s [45], matching the 2 s constant required for HD gain calibration. Synaptic plasticity is well known to depend on calcium, as well as on levels and timing of pre- and postsynaptic activity. Whilst no currently-known mechanism can account for the computation of the difference between average and instantaneous firing rates as required by Eqn 2, neurons are known to be capable of regulating their long term average firing rates to a set fixed point by increasing or decreasing total synaptic efficacies [46], and must therefore be able to calculate the difference between that set point and their long term averages. It is therefore not unreasonable to suggest that calculation of the difference of the decaying average and instantaneous firing rates is also possible by as yet unknown molecular mechanisms.

Known synaptic mechanisms could at least partially account for the required learning suppression effect, when synaptic updates for HD bump stability (condition 1) are suspended for a short period when the bump position is reset by landmark input. Inhibitory synaptic input is able to exert a shunting effect on an entire branch of a neuron's dendritic tree, effectively isolating that branch from the cell soma for the duration of the inhibitory current flow. It is conceivable (though is an untested hypothesis) that such shunting inhibition occurs in the HD network in mammals to restrict symmetric AHV input to the HD neurons during head direction resets. This hypothesis, and other suggestions above, are predictions of specific neuro-molecular mechanisms that may exist in the HD network in order for HD calibration to occur.

It is an open question how a turn gain calibration parameter could be stored and adjusted in the nervous system. The turn gain controls the overall efficacy of the synapses from the asymmetric AHV cells in the DTN to the HD cells in the LMN, or alternatively the overall firing rates of the AHV cells (see Figure 1). The control of turn gain could therefore manifest as a diffuse feedback effect from the HD cells to all of the asymmetric AHV cells in the DTN (or if not to DTN then to the population of AHV cells known to exist in LMN [9]). Diffuse effects are generally implemented in nervous systems through neuromodulatory signals. Neuromodulators are specialised neurotransmitters which, instead of undergoing immediate re-uptake at the synapse, diffuse through large areas of the brain and have an effect on multiple neurons. Our theory of 360° turn gain calibration therefore posits that HD cells should have a diffuse neuromodulatory effect on the strengths of all the connections from asymmetric AHV cells that synapse back to the HD cells themselves. This neuromodulation would be used to control the overall strength of the asymmetric AHV cells onto the HD cells, thereby controlling the speed of the HD bump. By this theory, any deficits in neuromodulation in these structures would result in deficits in calibration of the neural HD system. Dystonia (uncontrollable muscle movements resulting in awkward posture, gait and head and limb movements) is thought to be caused by disturbances in neuromodulator function. Interestingly, dystonic rats spend a much larger amount of time than normal controls in pivoting motions [47]; our theory of HD calibration would suggest this is due to sluggish calibration of the HD system, or perhaps complete calibration failure, resulting in repeated and prolonged attempts by the rat to recalibrate.

### Calibration of other movement systems

The head direction system is only one of the neural systems so far discovered which seem to contribute to animals' navigation abilities. Grid and place cells also hold allocentric (world-based) representations of an animal's position in its environment. Grid cells in particular, which fire in repeating triangular tessellations in all directions uniformly [48], are also likely to require calibration. Evidence for how grid cell firing patterns can be temporarily influenced by changing environmental cues before returning to their original configurations [49] suggests that some mechanism of calibration is likely to be active. Intriguingly, after the head turns and 360° pivoting warm-up behaviour displayed by rat pups and other infant mammals, periods of forward and backward rocking motions and even backwards walking can be observed [12]. We have demonstrated in the current study that head turns of alternating directions are useful for equalising turn speeds in the HDAAN. We conjecture that longitudinal rocking motions and backward walking could play a calibration role for translational motion tracking. Investigation of this potential mechanism for calibration of grid cell firing is left for future study.
